# The Effect of Plant Geographical Location and Developmental Stage on Root-Associated Microbiomes of *Gymnadenia conopsea*

**DOI:** 10.3389/fmicb.2020.01257

**Published:** 2020-06-18

**Authors:** Min Lin, Hui Xiong, Xuechuan Xiang, Zelin Zhou, Lifeng Liang, Zhinan Mei

**Affiliations:** ^1^School of Pharmaceutical Sciences, South-Central University for Nationalities, Wuhan, China; ^2^Institute of Ethnomedicine, South-Central University for Nationalities, Wuhan, China

**Keywords:** root-associated microbiomes, biogeography, plant compartment, plant growth stage, 16S rRNA gene, ITS2

## Abstract

*Gymnadenia conopsea* (L.) R. Br. is an important perennial terrestrial photosynthetic orchid species whose microbiomes are considered to play an important role in helping its germination and growth. However, the assemblage of *G. conopsea* root-associated microbial communities is poorly understood. The compositions of fungal and bacterial communities from the roots and corresponding soil samples in *G. conopsea* across distinct biogeographical regions from two significantly different altitudes were characterized at the vegetative and reproductive growth stages. The geographical location, developmental stage and compartment were factors contributing to microbiome variation in *G. conopsea*. Predominant fungal taxa include *Ascomycota*, *Basidiomycota*, *Mortierellomycota* and *Chytridiomycota*, whereas *Proteobacteria*, *Bacteroidetes*, *Acidobacteria*, *Actinobacteria*, *Verrucomicrobia*, *Chloroflexi*, *TM7* and *Planctomycetes* were predominant bacterial taxa. Using *G. conopsea* as a model, the structural and functional composition in *G. conopsea* root-associated microbiomes were comprehensive analyzed. Contrary to previous studies, biogeography was the main factor influencing the microbial community in this study. Besides, compartment and developmental stage should also be considered to analyze the variation of microbiota composition. Although the microbial composition varied greatly by location, the symbiotic microorganisms of *G. conopsea* still have certain specificity. This study gives an abundant information of *G. conopsea* root-associated microbiomes and provides new clues to better understanding the factors affecting the composition and diversity of fungal/bacterial communities associated with orchids. Our results also laying a foundation for harnessing the microbiome for sustainable *G. conopsea* cultivation. Moreover, these results might be generally applicable to other orchidaceae plants.

## Introduction

Terrestrial plants harbor abundant and diverse microbes that affect plant distribution, growth and health by different ways. Previous research revealed that plants can recruit beneficial/specific communities to cope with pathogen encounters, modify the nutrient status as well as adapt to new or changing environmental conditions (Qin et al., 2016; Berendsen et al., 2018; Yuan et al., 2018). In addition, the spatial distribution of soil microorganisms, such as microbial mutualists and pathogens, probably acts as a driver of spatial patterns of palnt species and plant community diversity (Ettema and Wardle, 2002). These communities can be taken as plant host’s second genome, providing a wide diversity of promising functional capacities (Berendsen et al., 2012; Turner et al., 2013). To maintain plant healthy growth, it is valuable to investigate the community structure of plant-associated microorganisms and their functions. So far, most research work has made on certain model and crop plants, such as *Arabidopsis* (Lundberg et al., 2012; Bai et al., 2015), rice (Sessitsch et al., 2012), maize (Peiffer et al., 2013), millet (Jin et al., 2017), *populus* (Cregger et al., 2018), and citrus (Xu et al., 2018), the results revealed clear taxonomically structured microbiome and the factors that determine assembly of the microbiome. This work may enable new plant management approaches and provide novel tools to improve the robustness of crop plant performance.

*Gymnadenia conopsea* (L.) R. Br. (Orchidaceae) is a terrestrial photosynthetic orchid species that widely distributed in Eurasia, including England, Ireland, Russia, Nepal, China, Japan, and the Korean peninsula (Hultén and Fries, 1986). *G. conopsea* exist in a wide range of habitats with an altitude ranging from 200 to 4700 m in China. For many years, *G. conopsea* was taken as a primary component in many preparations that listed in the Pharmacopoeia of the People’s Republic of China, and used for improving the body in traditional medicines, such as tonify Qi of the kidney and nourish the lungs and treat various diseases, especially in Tibetan medicine and Mongolian medicine. Whereas *G. conopsea* has been investigated as an important fragrant orchid plant in some European countries. *G. conopsea* is declining very rapidly like most other Orchidaceae. Now, *G. conopsea* was list in Convention on International Trade in Endangered Species of Wild Fauna and Flora (CITES). Two factors resulted in the rapid decline in the number of this species. The external factors were due to the high market demand, over exploitation as well as the habitat destruction, the internal endangered factors were its weak fertility and low breeding efficiency.

It was known that orchidaceae seeds are remarkably small, extremely light, and lacking endosperm (food reserves). Known seed weights extend from 0.31 to 24 μg, the diameter generally range from 0.01 to 0.9 mm, with the difference between the ‘widest’ and ‘thinnest’ known seeds in the family being 90-fold (Arditti and Ghani, 2000). Because orchidaceae seeds lack of endosperm, germination under natural conditions depend on some fungi colonization supplying carbohydrates (Smith and Read, 2008). In addition, Orchidaceae invariably rely on mycorrhizal fungi in their seedling and adulthood as well (Waterman et al., 2011; McCormick and Jacquemyn, 2014; Rasmussen et al., 2015). Early studies on the relationship between Orchidaceae plants and “orchid mycorrhiza” were mainly via germination experiments or cultivation methods in the laboratory conditions and found a diversity of associated fungi (Curtis, 1939). Though it is well-known that fungi have crucial roles in Orchidaceae plants, the effect of root-associated fungi on plant growth and distribution is less well-understood. Meanwhile, fungi cultivation under laboratory conditions does not reflect the complexity of interactions under natural conditions (Masuhara and Katsuya, 1994; Perkins et al., 1995). Furthermore, on account of their hidden lifestyle, a great deal of fungi cannot be cultivated under normal laboratory conditions, and it is difficult to guarantee the exist and diversity of fungi without contamination, or the contaminants may affects these early experimental results.

Amplicon based community profiling methods that amplify a wide taxonomic spectrum of fungi largely get rid of these effects as mentioned above and enable a direct evaluation of the fungal diversity in the orchid roots (Kristiansen et al., 2001; Taylor and McCormick, 2008; Voyron et al., 2017; McCormick et al., 2018). Previous molecular studies of the fungal communities associated with *G. conopsea* have indicated that this species is a mycorrhizal generalist, in that it can interact with a wide variety of fungi species (Stark et al., 2009; Jacquemyn et al., 2012; Tesitelova et al., 2013; Waud et al., 2016; Chen Y. et al., 2019). It mainly associated with the Tulasnellaceae and Ceratobasidiaceae (Tesitelova et al., 2013; Waud et al., 2016; Xing et al., 2020) and ectomycorrhizal fungi from Thelephoraceae, Russulaceae, Inocybaceae, and Cortinariaceae (Stark et al., 2009; Xing et al., 2020). Though the relevance and detail function of these fungi remain unknown, the available knowledge suggests that their exist have an ecological functions at least in certain other photosynthetic plant species (Jacquemyn et al., 2017). Moreover, the fungal community of *G. conopsea* demonstrated a obvious spatial structure, the mycorrhizal communities found in the roots of adult plants differ clearly between plants growing in different geographically distinct regions (Stark et al., 2009; Xing et al., 2020). Recent research in the seed germination experiments have revealed that *G. conopsea* is regarded a mycorrhizal generalist, however, it needs specific fungi to help protocorm formation and seedling development (Gao et al., 2020), it indicated the fungi of *G. conopsea* may vary among different developmental stage. Besides, previously investigations showed that bacteria isolated from orchid also benefit for plant growth and/or resistance to pathogens (Tsavkelova et al., 2007; Faria et al., 2013). Nevertheless, the information of the actual distribution and diversity of fungi in natural populations of *G. conopsea* in China are limited and how these fungi influence spatial and temporal patterns of recruitment and establishment of *G. conopsea* still unclear. Meanwhile, the composition structure and potentially functional activity of the *G. conopsea*-related bacteria remains largely unexplored.

In our study, the root and soil samples of the wild-grown *G. conopsea* plants from two different growth locations (habitats) and two developmental stages were collected. The dynamics of fungal and bacterial communities in *G. conopsea* of two distinct compartments (root and corresponding soil) from two distinct growth locations at two developmental stages were examined by ITS2 and 16S rRNA gene amplicon sequencing. Multiple factors contributing to microbial community variation were evaluated, and fungal and bacterial functions were predicted according to their taxonomy. Using the dynamic studies of the microbiome composition in large datasets from the different conditions sampled in this study, it offers new insights into the process of microbiome assembly in *G. conopsea*, and lays a foundation for sustainable use of the microbial community in *G. conopsea* cultivation.

## Materials and Methods

### Microbiome Sample Collection

Representative *G. conopsea* root samples and corresponding soil samples were collected uniformly from the natural habitats at Linzhi (29°38′59′′ N/94°25′37′′ E, 3600 m asl, Tibet, China) and Greater Khingan Mountains (51°42′N/124°20′E, 496 m asl, Heilongjiang, China). Linzhi (LZ) has a plateau temperate semi-humid and humid monsoon climate with annual mean rainfall 650 mm and annual mean temperature 8.7°C. Whereas, Greater Khingan Mountains (DXAL) belongs to the cold-temperate humid continental climate zone with annual mean rainfall 746 mm and annual mean temperature −2.8°C. The soil with alkali-hydrolyzale N 256.30/265.57 mg/kg, available P 48.00/27.76 mg/kg, available K 290.07/293.97 mg/kg; organic matter 121.67/66.55 g/kg, total N 4.58/5.92 g/kg, and a pH of 5.62/5.94 in LZ and DXAL, respectively. To rule out the effect of age on developmental stages, the plants with the consistent growth vigor at two developmental stages (vegetative growth stage and reproductive growth stage) were selected. The root and corresponding soil were sampled two times in LZ and DXAL, corresponding to two growth stages of plant. Plants were dug out with their surrounding soil, rapidly transferred into sterile plastic sampling bags and transported to the laboratory on ice as quickly as possible. The adjacent soil layers (about 1 cm thick) in the roots’ surface were manually separated and collected as the corresponding soil compartment. To remove fine roots and large organic debris, the soil samples were sieved through 2 mm mesh and are defined as the soil compartment. A total of 14 individual plants were collected in LZ and DXAL. The corresponding bulk soil of each plant were sampled five biological replicates. The roots were manually separated and then the adhering soil particles were removed by gently shaking. Five-seven roots (replicates) were used for each plant. The roots were cut into small pieces and washed thoroughly using pre-cooled sterile distilled water to take away the soil particles, and then washed successively with 75% pre-cooled ethanol, 0.25% NaOCl to further clean the root’ surfaces, and finally washed several times by sterile water. These step roots were defined as microbially-enriched root compartment. These treated soil and root samples were transferred to 2 mL sterile centrifuge tubes. All the samples were stored immediately at −80°C until subsequent use. In total, 70 soil samples and 75 root samples were obtained from two representative locations and two developmental stages in China.

### DNA Extraction and Sequencing for ITS2 and 16S rRNA Gene

Total DNA was extracted from aforementioned samples using the NucleoSpin® Soil Kit (Macherey-Nagel GmbH & Co. KG, Germany) according to the manufacturer’s instructions. The DNA quality and quantity were evaluated using the NanoDrop device (Thermo Fisher Scientific, United States) and agarose gel electrophoresis (1%). All the qualified DNA (total DNA concentration more than 50 ng and no protein, RNA and salt ion contamination) were used to construct library. All samples were carried out by sequencing the V3–V4 region of 16S rRNA gene in bacteria (341F-806R) and the ITS2 region of fungal nrDNA. These primers were designed to amplify target sequence and they include dual index and adapters for annealing to the Illumina Miseq flow cell. In both cases, only the qualified library can be used for sequencing. The qualified library was defined based on the following criteria: the amplified fragment length in the library consistent with expectation, total amount of PCR products ≥ 3 μg and concentration ≥ 30 ng/μ8L and agarose gel electrophoresis showed clear bands without dispersion. After DNA libraries quality control (QC) and quantification, as well as normalization, 300-bp paired-end reads were obtained from MiSeq platform (BGI, China) for the amplicon (16S rRNA and ITS2) analyses, respectively (Fadrosh et al., 2014).

### Amplicon Sequence Processing and Analyses

Fungal and bacterial community composition was detected by sequencing ITS2 and 16S rRNA gene amplicons from the *G. conopsea* root and corresponding soil samples. The high-quality illumina paired-end reads were merged using FLASH software with the default setting (Magoc and Salzberg, 2011). The operational taxonomic units (OTUs) were clustered using the UPARSE algorithm (Edgar, 2013) at 97% sequence identity, the chimeras were filtered by UCHIME (Edgar et al., 2011). To obtain the taxonomic information of the OTUs, the original reads were mapped back to their OTUs with the USEARCH (global alignment algorithm) (Edgar, 2010), the representative sequences in each OTU were obtained and mapped to Greengenes (v13_8 release/2013_5_99) and UNITE (2019_version8) reference databases by the Ribosomal Database Project classifier (Wang et al., 2007) for the bacterial and fungal communities, respectively. OTUs classified as mitochondria or chloroplast, or less than 5 sequences, were filtered from the datasets.

### Diversity Analysis

The alpha-diversity was computed for each sample by Chao and Shannon indices using mothur (Schloss et al., 2009) from the final OTU table. Boxplot were plotted using R software. The significant differences in alpha-diversity from different groups were determined by the Kruskal–Wallis and Dunn’s *post hoc* tests (*P* < 0.05). The OTU table was normalized by the cumulative sum scaling (CSS) method to calculate beta-diversity (Paulson et al., 2013). Unweighted UniFrac (UUF) and Weighted UniFrac (WUF) distances between samples were computed from the above-mentioned normalized OTU table. The taxonomic dissimilarity analyses between samples was performed based on principal coordinate analysis (PCoA) based on WUF and UUF. To assess the influence of the different factors (site, compartment, and stage) on the beta-diversity, permutational multivariate analysis of variance (PERMANOVA) analysis was carried out (adonis function in vegan R package).

### Significantly Differential Core OTUs Analysis

Operational taxonomic units with frequency of occurrence above 90% from the root or soil samples in each group were defined as “core OTUs.” To identify significantly differential core OTUs between each pair of groups that need to compare, the Wilcoxon signed rank test was employed in each group, and with relative abundance (RA) considered separately in each sample. The resulting *P*-values were corrected for multiple testing via the Benjamini-Hochberg (BH) method. The core OTUs enriched in each group (*P* < 0.05) were then referred to as “differential core OTUs.” The comparison of core taxa of the root/soil microbiome between each pair of groups were based on differential core OTUs.

### Microbial Function Prediction

In order to confirm if fungal functional groups differed in relative abundance between host biogeography within compartment (root, soil) at two developmental stages, the FUNGuild (Nguyen et al., 2016) was used to classify each core OTU into an ecological guild. Core OTUs assigned to a guild with the confidence ranking of “probable” or “highly probable” were retained for further use, whereas those with “possible” were removed. Bacteria microbial function prediction was carried out by the PICRUSt software. Differentially enriched KO pathways or modules were identified on the basis of the reporter score from Z-scores of individual KOs. One tail Wilcoxon rank sum test was applied to all Kos, and corrected for multiple testing by the Benjamin-Hochberg method. The Z-score of each KO could then be computed. The detection threshold of significantly differentiating pathways was set as absolute value for reporter score ≥ 1.96 (95% confidence in either tail basing on normal distribution).

## Results

### Root-Associated Microbial Assemblages

**FIGURE 1:**
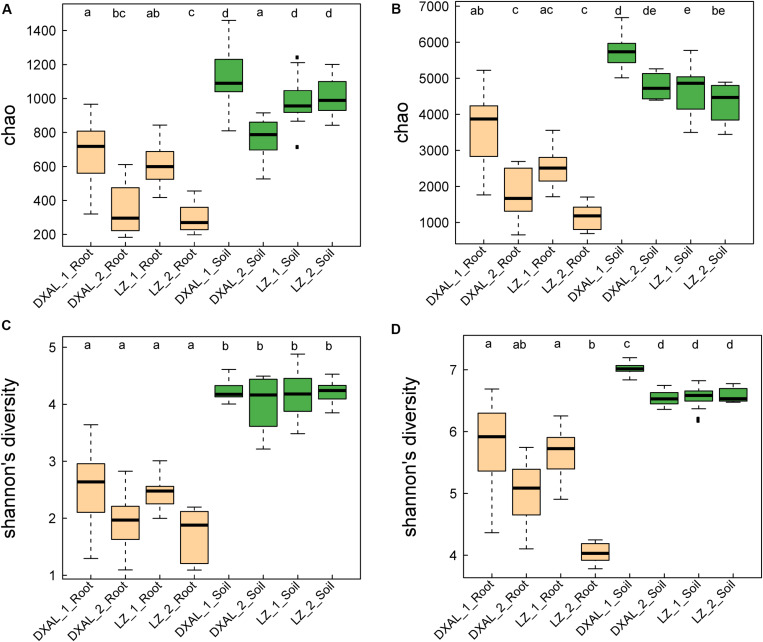
Species richness and diversity of root-associated microbial communities. **(A)** Microbial richness index comparison between each group based on the Chao index using the ITS data. **(B)** Microbial diversity index comparison between each group based on the Shannon index using the ITS data. **(C)** Microbial richness index comparison between each group based on the Chao index using the 16S rRNA gene data. **(D)** Microbial diversity index comparison between each group based on the Shannon index using the 16S rRNA gene data. Significant differences are depicted with letters (*P* < 0.05, Kruskal–Wallis with Dunn’s *post hoc* test). DXAL: Greater Khingan Mountains, LZ: Linzhi, 1: vegetative growth stage, 2: reproductive growth stage.

We analyzed fungal and bacterial microbiomes from two separate rhizocompartments (the root and corresponding soil), a total of 145 samples that included 69 samples from LZ and 76 samples from DXAL (Supplementary Table S1). The ITS2 and V3–V4 region of 16S rRNA gene were amplified by PCR and sequenced by Illumina MiSeq platform. Approximately 12.1 and 5.1 million high-quality sequences were obtained from the ITS2 and 16S rRNA gene sequencing data, respectively. 6,667,165 and 5,460,693 ITS2 tags and 3,883,175 and 1,206,448 16S rRNA tags were obtained from the corresponding soil and root sample, respectively (Supplementary Tables S2, S3). After discarding non-bacterial or non-fungi, mitochondrial, chloroplast and low-abundance OTUs, we obtained 5,193 fungi OTUs and 9,712 bacterial OTUs, respectively (Supplementary Tables S2, S3). For the microbial richness Chao index estimated based on the fungal dataset, it revealed that the microbial richness from soil to the root was decreased in both sites (Figure 1A, *P* < 0.05), except the soil sample in DXAL at reproductive growth stage. Significant differences were found in comparisons between two different developmental stages in root samples include LZ and DXAL, the richness value was lower in reproductive growth stage than the vegetative growth stage. Similar results were retrieved from microbial diversity shannon index values, with higher diversity in soil samples (Figure 1B, *P* < 0.05). For the bacterial microbiomes, measures of within-sample diversity (α-diversity indices) showed a decrease of microbial richness and diversity from soil samples to root samples based on the chao index and shannon index in both sites (Figures 1C,D, *P* < 0.05). In both bacterial microbial richness and diversity, higher richness and diversity were observed in vegetative growth stage than reproductive growth stage at two sides (*P* < 0.05).

**FIGURE 2:**
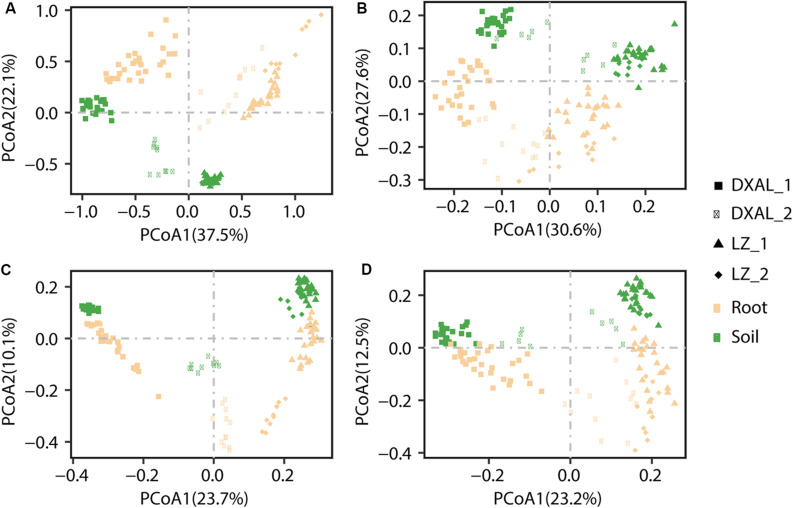
PCoA of microbial community composition among different sample types of *G. conopsea*. **(A)** PCoA using the WUF distances at the OTU level using fungal community’s data. **(B)** PCoA using the UUF distances at the OTU level using fungal community’s data. **(C)** PCoA using the WUF distances at the OTU level using bacterial community’s data. **(D)** PCoA using the UUF distances at the OTU level using bacterial community’s data. Samples are color coded according to the sites, compartments, and developmental stages are depicted with different symbols. DXAL: Greater Khingan Mountains, LZ: Linzhi, 1: vegetative growth stage, 2: reproductive growth stage.

To examine between-sample variation (β-diversity) and to investigate patterns of separation between microbial communities, PCoAs based on WUF and UUF distances were performed. In both the WUF and UUF PCoAs, fungal and bacterial communities cluster along the first principal coordinate based on the geographical location and development stage, the second factor explaining fungal and bacterial communities was slightly different (Figure 2A; WUF, 2B UUF; 2C WUF, 2D, UUF). In fungal community, compartment was the second factor using WUF metric, while not obvious by UUF metric (Figure 2B); however, in bacterial community, compartment was the second factor in all metric (Figures 2C,D). Interestingly, the PCoA result of the bacterial communities, some samples (include root and soil samples) in the DXAL at reproductive growth stage were close to LZ samples. Additionally, the data for clustering of samples were computed by PERMANOVA with all distance, which revealed that site, compartment and developmental stage comprise the largest contribution to variation for the microbiome data in both fungal and bacterial communities, it was consistent with the PCoA results [R2 of 0.709 and 0.644 (WUF); 0.462 and 0.429 (UUF), respectively, *P*-value < 0.001 for all tests; Supplementary Table S4]. Whereas according to the single factor, site was the strongest factor shaping these microbiome communities, followed by compartment or plant development stage [R2 of 0.272, 0.204, and 0.091 (WUF); 0.203, 0.063, and 0.072 (UUF); 0.303, 0.076 in fungal community; R2 of 0.244, 0.226, and 0.078 (WUF); 0.177, 0.089, and 0.059 (UUF) in bacterial community, for site, compartment, and stage, respectively, *P*-value < 0.001 for all tests; Supplementary Table S4]. Together, these results implying that microbial communities vary significantly between plant host biogeography, compartments and developmental stages also impact *G. conopsea* microbial community composition.

### Taxonomic Assignment of Microbial Community Composition

**FIGURE 3:**
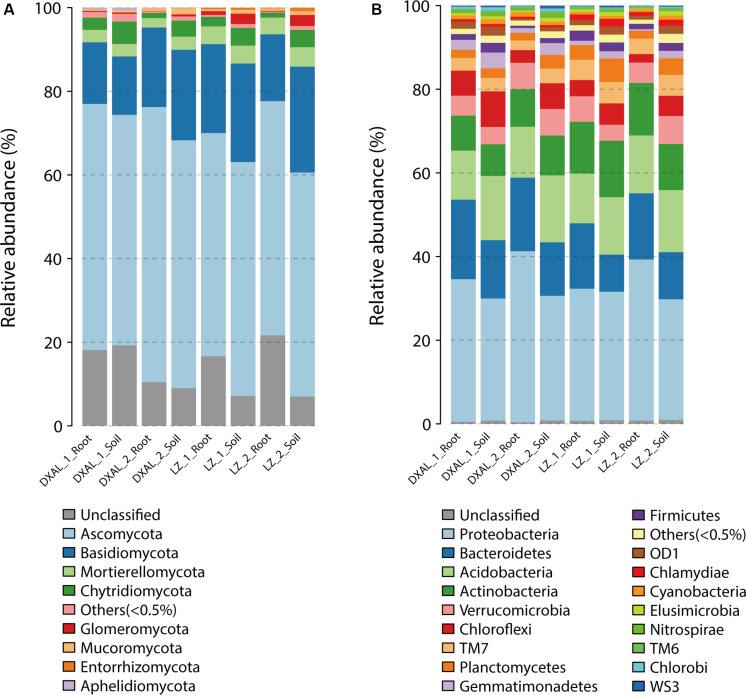
Microbial community composition among different sample types of *G. conopsea* at phylum level. **(A)** Relative abundance of fungal taxa of each group. **(B)** Relative abundance of bacterial taxa of each group. DXAL: Greater Khingan Mountains, LZ: Linzhi, 1: vegetative growth stage, 2: reproductive growth stage.

A totally of 17/39 different phyla and 65/122 different classes were identified in fungal/bacterial communities, respectively. As expected, there were obvious differences in the proportions of various phyla and classes among the compartments that are consistent with each site and development stage (Figure 3 and Supplementary Figure S1). The dominant (RA > 1%) fungal phyla found in the samples included *Ascomycota* (>50%), *Basidiomycota*, *Mortierellomycota* and *Chytridiomycota*, whereas prokaryotic phyla, included *Proteobacteria* (>28%), *Bacteroidetes*, *Acidobacteria*, *Actinobacteria*, *Verrucomicrobia*, *Chloroflexi*, *TM7* and *Planctomycetes* (Supplementary Table S5). At lower taxonomic ranks, *Leotiomycetes*, *Agaricomycetes*, *Dothideomycetes*, *Sordariomycetes*, *Eurotiomycetes*, *Tremellomycetes*, and *Mortierellomycetes* were the dominant fungal classes found in both compartments across two sites, *Alphaproteobacteria*, *Betaproteobacteria*, *Deltaproteobacteria*, *Saprospirae*, *Gammaproteobacteria*, *Sphingobacteriia*, *Actinobacteria*, *Acidobacteria-6*, *Thermoleophilia*, *TM7-1*, *Acidimicrobiia*, *Solibacteres*, *Cytophagia*, and *Spartobacteria* were the main bacterial classes (Supplementary Table S6). The root had a significantly greater proportion of *Leotiomycetes* and *Tremellomycetes* (*q*-value < 0.05) than soil, whereas *Agaricomycetes*, *Glomeromycetes*, *Lobulomycetes*, *Rhizophydiomycetes*, and *Spizellomycetes* (*q*-value < 0.05) were largely depleted in the root in comparison with the soil in fungal communities (Supplementary Table S7). In the bacterial communities, *Alphaproteobacteria*, Methylacidiphilae, *Sphingobacteriia* and *TM7_3* (*q*-value < 0.01) were more abundant in root, while *Acidobacteria_6*, *AT_s54*, *EC1113*, *Gemm_1*, *Gemm_2*, *Gemm_5*, *Gemmatimonadetes*, *iii1_8*, *ML635J_21*, *Nitrospira*, *OM190*, *Phycisphaerae*, *PRR_12*, *RB25*, *S035*, *S085*, *SJA_28*, *SM2F11*, *TA18*, *TK10*, *TK17*, and *TM1* (*q*-value < 0.01) was more prevalent in soil (Supplementary Table S8). The decrease in relative abundance of these classes among compartments was coincident with the detection that the microbial diversity reduced from soil to root.

### Core Functional Prediction of the Microbiome

**FIGURE 4:**
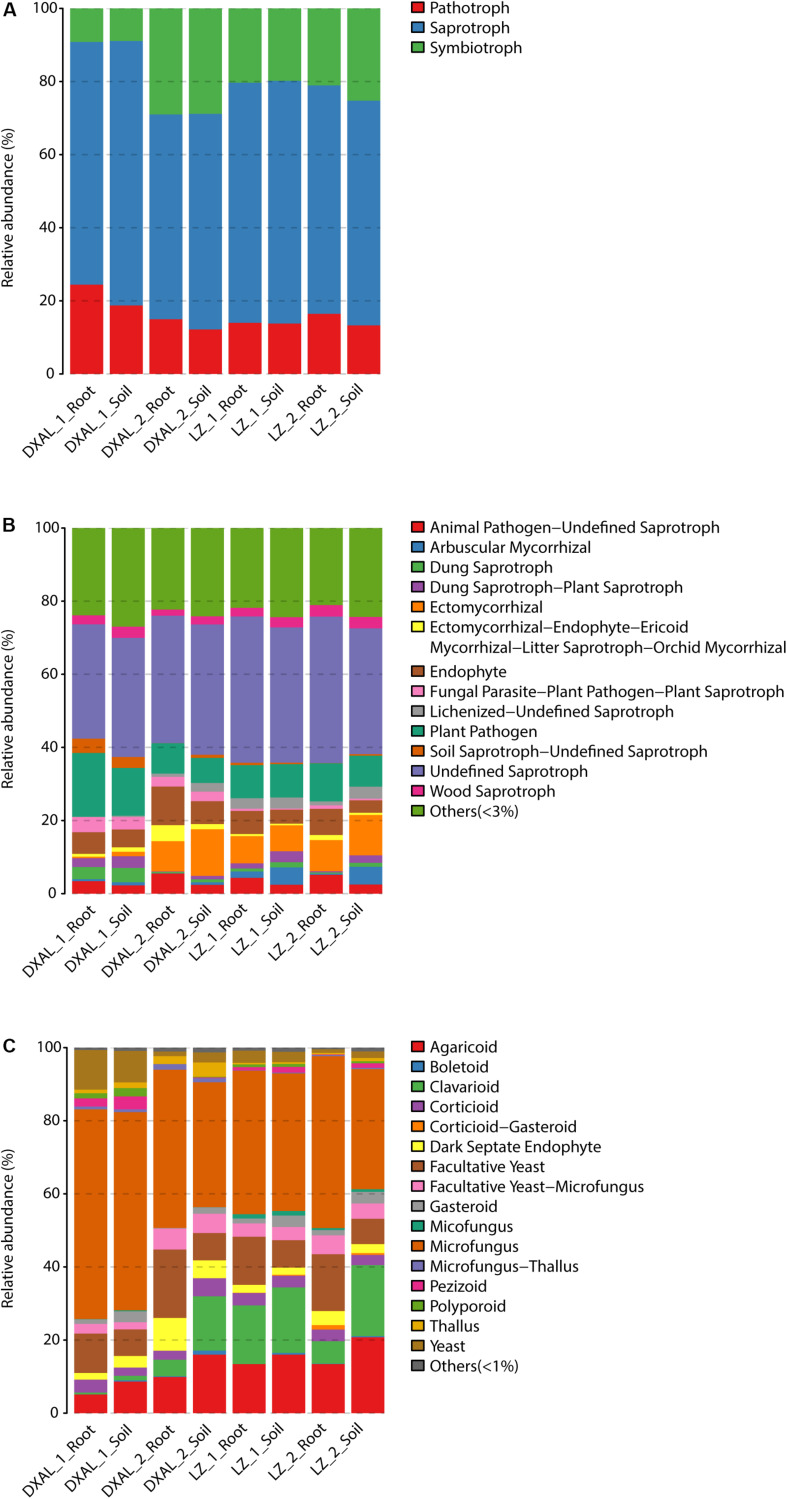
Compositions of fungal functional group (guild) inferred by FUNGuild. **(A)** At the trophic mode level. **(B)** At guilds level. **(C)** At Growth_Morphology level. DXAL: Greater Khingan Mountains, LZ: Linzhi, 1: vegetative growth stage, 2: reproductive growth stage.

As microbes that are consistently present across samples likely provide critical ecological functions (Shade and Handelsman, 2012), the core fungal/bacterial communities were analyzed in each group. We defined 390/718 core root fungal OTUs and 1190/1314 core soil fungal OTUs from LZ and DXAL microbiota throughout the two growth stages, respectively. Regarding fungal functional group (guild), all core OTU were classify based on FunGuild assignment. Since the whole genome information of fungi is still relatively scarce, the functional annotation information of fungi is less comprehensive. There were only 38.24% (910/2380) core OTU have reliable functional annotation information. From the trophic mode level, saprotroph was most dominant trophic mode, followed by symbiotroph and pathotroph (Figure 4A and Supplementary Table S9). Undefined_Saprotroph, Plant_Pathogen, Endophyte, Ectomycorrhizal and Animal_Pathogen-Undefined_Saprotroph were the top five high-abundance guilds (Figure 4B and Supplementary Table S9). The Growth_Morphology mainly focus on Microfungus, Agaricoid, Facultative_Yeast, Clavarioid, Yeast, Facultative_Yeast-Microfungus, Dark_Septate_Endophyte, Corticioid, Gasteroid, Thallus, and Pezizoid (Figure 4C and Supplementary Table S9).

**FIGURE 5:**
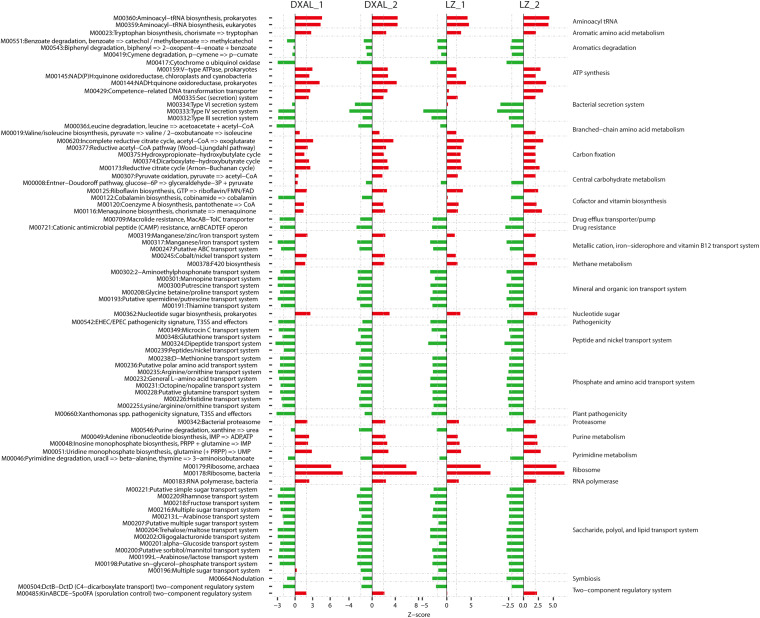
Alterations in bacteria microbial functional modules in each compare group. Dashed lines indicate a reporter score of 1.96, corresponding to 95% confidence in a normal distribution. DXAL_1: DXAL_1_Root vs. DXAL_1_Soil, DXAL_2: DXAL_2_Root vs. DXAL_2_Soil, LZ_1: LZ_1_Root vs. LZ_1_Soil, LZ_2: LZ_2_Root vs. LZ_2_Soil. DXAL: Greater Khingan Mountains, LZ: Linzhi, 1: vegetative growth stage, 2: reproductive growth stage.

For the bacterial core OTUs, 1,417/3,099 core root OTUs and 4,403/5,920 core soil OTUs from LZ and DXAL microbiota were obtained throughout the two growth stages, respectively, using the aforementioned method. In order to gain insight into functional changes within *G. conopsea* bacterial microbiota, the KEGG pathways and modules enriched in root or soil microbiomes were analyzed (Figure 5 and Supplementary Table S10). The samples from root displayed higher potential for plant nutrition, such as transfer of amino acids and sugars, stress resistance, Aromatics degradation, Drug efflux transporter/pump, Drug resistance, Mineral and organic ion transport system, Pathogenicity, Peptide and nickel transport system, Plant pathogenicity, Phosphate and amino acid transport system, Saccharide, polyol, and lipid transport system and Symbiosis, whereas the modules for Energy metabolism, Nucleotide and amino acid metabolism and Genetic information processing (Aminoacyl tRNA, Aromatic amino acid metabolism, ATP synthesis, Carbon fixation, Cofactor and vitamin biosynthesis, Methane metabolism, Nucleotide sugar, Proteasome, Ribosome and RNA polymerase) were enriched in soil samples (Figure 5 and Supplementary Table S10). Nutrition is of great importance in shaping the root microbiota, the root containing various plant-derived compounds that would be likely source of nutrients for microbes. Consistent with this, the modules that including transport system responsible for transferring phosphate, amino acid, peptide, nickel, Saccharide, polyol, and lipid were enriched in the core root microbiome. Symbiosis is widely thought to act an important role in plant growth owing to its function in plant biodiversity and nutrient supply (van der Heijden et al., 2016), and was observed significantly enriched in root samples compared with soil (Figure 5). The soil microbiome exhibited increased potential for Carbon fixation, consistent with the enrichment of *Cyanobacteria* in soil samples (Supplementary Table S5).

### Comparative Analyses of Core Microbiome Across Different Sites

**FIGURE 6:**
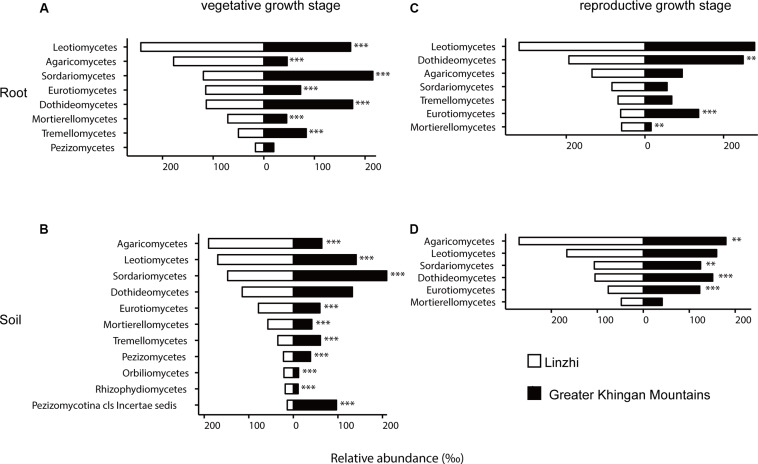
Side-by-side comparison of the relative abundance (‰) of classes which made up at least > 10‰ of the total fungal core microbiome community across different sites. **(A)** Relative abundance (‰) of the classes detected in root core OTUs of the indicated plant growth sites at vegetative growth stage. **(B)** Relative abundance (‰) of the classes detected in soil core OTUs of the indicated plant growth sites at vegetative growth stage. **(C)** Relative abundance (‰) of the classes detected in root core OTUs of the indicated plant growth sites at reproductive growth stage. **(D)** Relative abundance (‰) of the classes detected in soil core OTUs of the indicated plant growth sites at reproductive growth stage. Asterisks indicate significant differences occur between Linzhi and Greater Khingan Mountains [Benjamini–Hochberg false discovery-rate (FDR) adjusted *P*-value, ^∗^ represent < 0.05, ^∗∗^ represent < 0.01, ^∗∗∗^ represent < 0.001] in each compartment.

To investigate how plant geographical locations might affect the microbiome, the core OTU that significantly different in abundance in each group were used for further analysis, and the fungal and bacterial community compositions from two plants growth locations were compared (Supplementary Tables S11, S12). The fungal community composition was notably different between two sites at the vegetative growth stage in both root and soil samples, *Leotiomycetes*, *Agaricomycetes*, *Eurotiomycetes*, and *Mortierellomycetes* were more abundant in LZ core fungal OTUs, whereas *Sordariomycetes*, *Dothideomycetes*, and *Tremellomycetes* were more prevalent in DXAL core fungal OTUs (Figures 6A,B, *P <* 0.05). The fungal community compositions among samples at the reproductive growth stage were more similar in two sites. In the root samples, *Mortierellomycetes* was more abundant in LZ, whereas *Dothideomycetes* and *Eurotiomycetes* were more prevalent in DXAL (Figure 6C), as for the soil samples, the relative abundance of *Sordariomycetes*, *Dothideomycetes* and *Eurotiomycetes* were higher in DXAL, whereas that of *Agaricomycetes* was lower in DXAL (Figure 6D).

**FIGURE 7:**
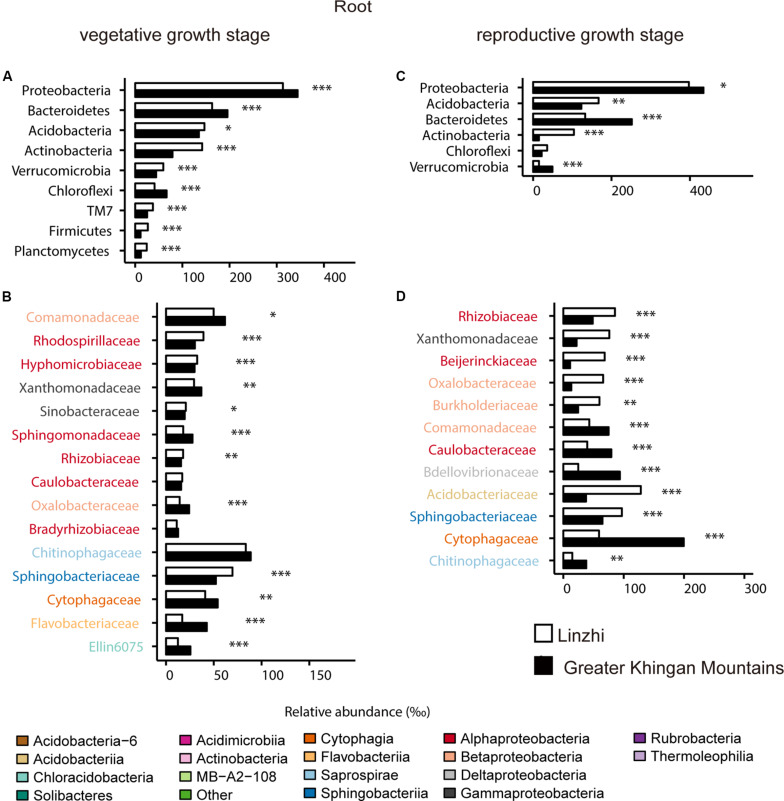
Comparative analyses of bacterial core microbiome across different sites. **(A)** Relative abundance (‰) of the phyla detected in root core OTUs of the indicated plant growth sites at vegetative growth stage. **(B)** Relative abundance (‰) of families belonging to the three dominant phyla in the root core OTUs of the indicated plant growth sites at vegetative growth stage. **(C)** Relative abundance (‰) of the phyla detected in root core OTUs of the indicated plant growth sites at reproductive growth stage. **(D)** Relative abundance (‰) of families belonging to the three dominant phyla in the root core OTUs of the indicated plant growth sites at reproductive growth stage. Asterisks indicate significant differences [Benjamini–Hochberg false discovery-rate (FDR) adjusted *P*-value, ^∗^ represent < 0.05, ^∗∗^ represent < 0.01, ^∗∗∗^ represent < 0.001].

For the taxonomic structure in the bacterial communities, *Proteobacteria*, *Bacteroidetes*, *Acidobacteria*, *Actinobacteria*, *Verrucomicrobia*, *Chloroflexi*, *TM7*, *Firmicutes*, and *Planctomycetes* as dominating phyla in root bacterial communities at the vegetative growth stage, these phyla in relative abundance were significantly different between two sites (Figure 7A). The families belonging to the three dominant (*Proteobacteria*, *Bacteroidetes*, and *Acidobacteria*) phyla were listed, which contained 15 families and belonged to 8 classes (Figure 7B). *Rhodospirillaceae*, *Hyphomicrobiaceae* and *Rhizobiaceae* (*Alphaproteobacteria*), *Sinobacteraceae* (*Gammaproteobacteria*), *Sphingomonadaceae* (*Bacteroidetes*) are significantly enriched in LZ compared to DXAL at the vegetative growth stage, whereas *Comamonadaceae* and *Oxalobacteraceae* (*Betaproteobacteria*), *Xanthomonadaceae* (*Gammaproteobacteria*), *Sphingomonada ceae* (*Alphaproteobacteria*), *Cytophagaceae* (*Cytophagia*), *Flavobacteriaceae* (*Flavobacteriia*), *Ellin6075* (*Chloracidobacteria*) were more abundant in DXAL (Figure 7B). As for the reproductive growth stage, the relative abundance in *Proteobacteria*, *Acidobacteria*, *Bacteroidetes*, *Actinobacteria* and *Verrucomicrobia* were significantly different between two sites (Figure 7C). We noted that most classes belonging to the *Proteobacteria*, were over-representation in LZ, such as *Rhizobiaceae* and *Beijerinckiaceae* (*Alphaproteobacteria*), *Xanthomonadaceae* (*Gammaproteobacteria*), *Oxalobacteraceae* and *Burkholderiaceae* (*Betaproteobacteria*), besides, *Acidobacteria*ceae (*Acidobacteriia*), and *Sphingobacteriaceae* (*Sphingobacteriia*) also more dominated in LZ; whereas *Comamonadaceae* (*Betaproteobacteria*), *Caulobacteraceae* (*Alphaproteobacteria*), *Bdellovibrionaceae* (*Deltaproteobacteria*), *Cytophagaceae* (*Cytophagia*), *Chitinophagaceae* (*Saprospirae*) were significantly enriched in DXAL (Figure 7D).

Likewise, for the soil samples, the difference of bacterial community composition between two sites was shown in Supplementary Figure S2 and Supplementary Table S12. At vegetative growth stage, the dominant phyla were *Proteobacteria*, *Actinobacteria*, and *Acidobacteria* (Supplementary Figure S2A). *Rhodospirillaceae* (*Alphaproteobacteria*), *Sinobacteraceae* (*Gammaproteobacteria*), *Gaiellaceae* (*Thermoleophilia*), *EB1017* and *C111* (*Acidimicrobiia*), *RB40* (*Acidobacteria-6*) were significantly enriched in LZ, whereas *Syntrophobacteraceae* and *Bdellovibrionaceae* (*Deltaproteobacteria*), *Sphingomonadaceae* (*Alphaproteobacteria*), *Ellin6075* (*Chloracidobacteria*) were over-present in DXAL (Supplementary Figure S2B); As for the reproductive growth stage, the different phyla was similar with vegetative growth stage (Supplementary Figure S2C), at the family level, the significantly different families were less, *Sinobacteraceae* and *Coxiellaceae* (*Gammaproteobacteria*), *Koribacteraceae* (*Acidobacteriia*), *Solibacteraceae* (*Solibacteres*), *RB40* (*Acidobacteria-6*), *EB1017* (*Acidimicrobiia*), *Nocardioidaceae* (*Actinobacteria*) were dominant families in LZ soil bacterial core OTUs, only *Gaiellaceae* (*Thermoleophilia*) was significantly more enriched in DXAL (Supplementary Figure S2D). These results showed microbial composition differed between host biogeography, and the common fungal and bacterial taxa were seen among each location that were roughly in line with other plants (Ottesen et al., 2013; Pinto et al., 2014; Peay et al., 2016).

### Comparative Analyses of Core Microbiome Across Plant Growth Stages

**FIGURE 8:**
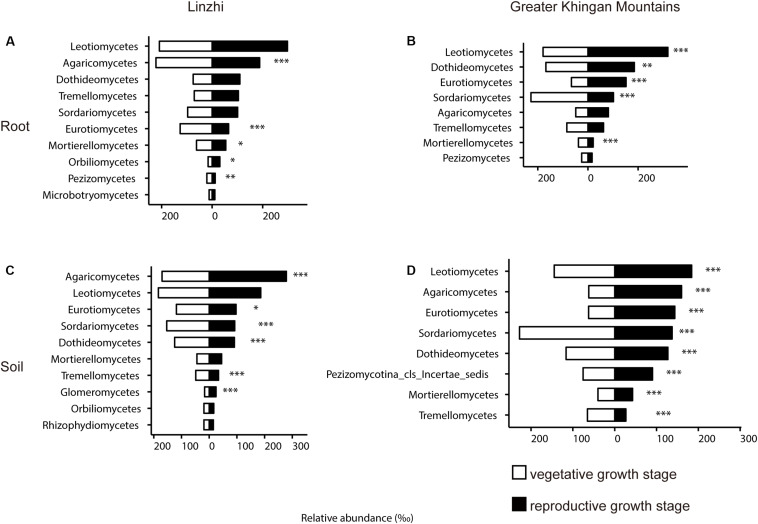
Side-by-side comparison of the relative abundance (‰) of classes which made up at least > 10‰ of the total fungal core microbiome community across different developmental stages. **(A)** Relative abundance (‰) of the classes detected in root core OTUs of the indicated developmental stages in Linzhi. **(B)** Relative abundance (‰) of the classes detected in soil core OTUs of the indicated plant growth sites in Linzhi. **(C)** Relative abundance (‰) of the classes detected in root core OTUs of the indicated plant growth sites in Greater Khingan Mountains. **(D)** Relative abundance (‰) of the classes detected in soil core OTUs of the indicated plant growth sites in Greater Khingan Mountains. Asterisks indicate significant differences occur between vegetative growth stage and reproductive growth stage [Benjamini–Hochberg false discovery-rate (FDR) adjusted *P*-value, ^∗^ represent < 0.05, ^∗∗^ represent < 0.01, ^∗∗∗^ represent < 0.001] in each compartment.

The previous study revealed that the root-associated microbiomes has been proven to be associated with the plant developmental stages (Lundberg et al., 2012; Skaltsas et al., 2019). To investigate the relationship between growth stage and the root-associated microbiome, two growth stages in two different growing regions were tested and compared. The difference in community composition of fungi at the class level in the root and soil from two sites under two growth stages are shown in Figure 8 and Supplementary Table S13. In LZ, distribution of taxonomic classes between vegetative growth stage and reproductive growth stage were overall similar: *Leotiomycetes* and *Agaricomycetes* make up the majority of the plant microbiota. The distribution of *Agaricomycetes*, *Eurotiomycetes*, *Mortierellomycetes*, and *Pezizomycetes* significantly showed a decreasing trend from vegetative growth stage to reproductive growth stage among root samples, only *Orbiliomycetes* was showed an increasing trend (Figure 8A). *Eurotiomycetes*, *Sordariomycetes*, *Dothideomycetes*, and *Tremellomycetes* were enriched at vegetative growth stage in the soil compartment, while *Agaricomycetes*, *Glomeromycetes*, and *Mortierellomycetes* were enriched at reproductive growth stage (Figure 8B). As for DXAL, *Sordariomycetes* and *Mortierellomycetes* were over-present in vegetative growth stage in the root compartment, whereas *Leotiomycetes*, *Dothideomycetes*, and *Eurotiomycetes* were over-present in reproductive growth stage (Figure 8C). *Sordariomycetes* and *Tremellomycetes* were more dominant in vegetative growth stage in the soil compartment, whereas *Leotiomycetes*, *Agaricomycetes*, *Eurotiomycetes*, *Dothideomycetes*, and *Mortierellomycetes* were highly persistent in reproductive growth stage (Figure 8D).

**FIGURE 9:**
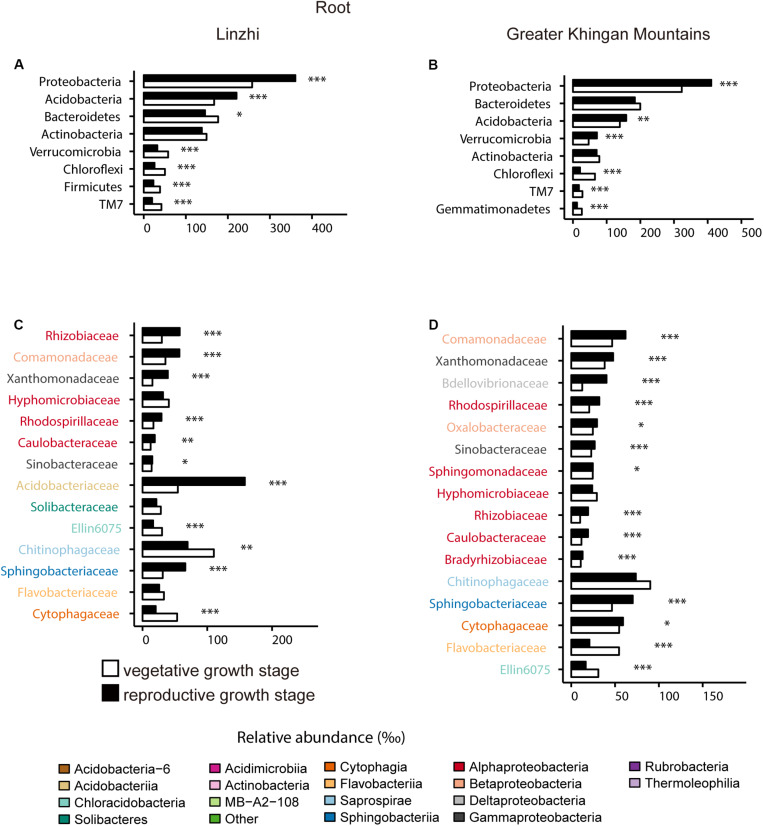
Comparative analyses of bacterial core microbiome across different developmental stages. **(A)** Relative abundance (‰) of the phyla detected in root core OTUs of the indicated developmental stages in Linzhi. **(B)** Relative abundance (‰) of families belonging to the three dominant phyla in the root core OTUs of the indicated developmental stages in Linzhi. **(C)** Relative abundance (‰) of the phyla detected in root core OTUs of the indicated plant growth sites in Greater Khingan Mountains. **(D)** Relative abundance (‰) of families belonging to the three dominant phyla in the root core OTUs of the indicated plant growth sites in Greater Khingan Mountains. Asterisks indicate significant differences [Benjamini–Hochberg false discovery-rate (FDR) adjusted *P*-value, ^∗^ represent < 0.05, ^∗∗^ represent < 0.01, ^∗∗∗^ represent < 0.001].

For the difference of bacterial community composition between growth stage at two sites was shown in Figure 9, Supplementary Figure S3 and Supplementary Table S14, the differential core OTUs involved phyla are almost similar at two sites in the root compartment, such as *Proteobacteria*, *Acidobacteria*, *Verrucomicrobia*, *Chloroflexi* and *TM7*, except *Bacteroidetes* and *Firmicutes* in LZ, and *Gemmatimonadetes* in DXAL (Figures 9A,C). These dominate phyla were further analyzed at the family level. At root samples, whether in LZ or DXAL, relative abundance of most families was higher in reproductive growth stage, for example, *Rhizobiaceae*, *Rhodospirillaceae* and *Caulobacteraceae* (*Alphaproteobacteria*), *Xanthomonadaceae* and *Sinobacteraceae* (*Gammaproteobacteria*), *Comamonadaceae* (*Betaproteobacteria*), *Sphingobacteriaceae* (*Sphingobacteriia*), at the vegetative growth stage, *Hyphomicrobiaceae* (*Alphaproteobacteria*), *Ellin6075* (*Chloracidobacteria*), *Chitinophagaceae* (*Saprospirae*), *Cytophagaceae* (*Cytophagia*) were more abundant in LZ (Figure 9B), and *Flavobacteriaceae* (*Flavobacteriia*), *Ellin6075* (*Chloracidobacteria*) were more prevent in DXAL (Figure 9D). For the soil samples, the differential core OTUs involved phyla were similar like root samples, but the significantly enriched families in three dominant phyla were different from root. Whether in LZ or DXAL, the relative abundance of most families was higher in the vegetative growth stage (Supplementary Figure S3 and Supplementary Table S14). Consistent with previous observations, microbial composition differed between different development stages, it suggested that the overall recruitment of OTUs is governed by a set of factors that include developmental stage.

## Discussion

In the present study, we performed geographical location and developmental stage investigation on the taxonomic characters of *G. conopsea* root-associated microbiomes to gain deeper insight into plant driven taxa and their features in each habitat. Earlier researches on root microbiome have mainly focused on other orchids (Leake and Cameron, 2012; Selosse and Martos, 2014; Xing et al., 2015; Fochi et al., 2017; Voyron et al., 2017). Due to the decreased cost of amplicon-based sequencing, it makes the studies of microbiomes in large-scale and global possible. Recently, Chen and colleagues analyzed the root and rhizosphere microbiomes in wheat by combining the effect of developmental stages and nitrogen fertilization using a amplicon sequencing approach, their results implied that both developmental stages and nitrogen input affect the structure of root microbial community in wheat (Chen S. et al., 2019). Here, we analyzed the factors driving changes in the composition of root-associated microbial community structure using amplicon sequencing approach. This is the first time that simultaneously examine the root-associated microbiomes that includes both fungi and bacteria in *G. conopsea*.

As revealed by the alpha-diversity index, there was a significant difference in species diversity between corresponding soil and root in *G. conopsea*, it is consistent with the universal reports of microbial colonization (Beckers et al., 2017; Duran et al., 2018). Soil serves as one of the richest microbial ecosystems on Earth, providing ideal habitats for various microbial lineages (Wei and Ashman, 2018). The species diversity of plant compartments (root) was significantly lower, mainly owing to that, the colonization pattern and formation of a stable microbiome within root were more complicated than expected, such as the response of plant’s the innate immune system to microbial colonization. The decrease of species diversity and richness from soil to plant root showed that only a small part of fungal/bacterial microbes could maintain symbiotic associations with its host plant and ultimately form dominant microbial assemblages in root.

According to the PCoA analysis, notably variation in structure of fungal/bacterial communities was further uncovered regarding compartment, plant geographical location and developmental stage. The results revealed that the most important factor determining fungal/bacterial microbiome dynamics in *G. conopsea* were the geographical locations of plant. This pattern was in agreement with PERMANOVA results according to UniFrac distances. Our result was consistent with the findings reported by Duran et al. (2018), in which the host biogeography was the key factor shaping the composition of root fungal community. It was also in accord with previous study that a general increase in differentiation with increasing geographical distance in *G. conopse*-associated fungal communities (Stark et al., 2009; Xing et al., 2020). However, the result of bacterial microbiome was different from the study of other plant, such as rice (Edwards et al., 2015), *Arabidopsis* (Duran et al., 2018), millet (Jin et al., 2017), *Cycas panzhihuaensis* (Zheng and Gong, 2019), which suggested that the compartment was the main factor shaping the composition of the bacterial microbiome. It may relate to the growth characteristics of orchids, which fully or partially relies on mycorrhizal fungi for requisite nutrients; meanwhile, some bacteria, such as the mycorrhiza helper bacteria (MHB) were found to be commonly occurring in ectomycorrhiza and in arbuscular mycorriza associations, which assist mycorrhiza formation (Frey-Klett and Garbaye, 2005; Faria et al., 2013; Esposito-Polesi et al., 2017; Li et al., 2017). Nonetheless, very little is known about the composition, structure, and functional activity of bacterial microbiome associated with orchid, let alone the information in *G. conopsea*. Thus, the major factors affecting the composition of bacterial community in *G. conopsea* requires further study. In general, these results indicated that *G. conopse* are not specialists and have a significant amount of geographical variance in their fungal/bacterial communities.

In our study, the samples were derived from two different altitude places approximately 5,466 kilometers away. LZ is in the southern part of Tibet, the altitude is 3600 m, and has a semi-humid tropical climate, whereas DXAL belongs to the north of Heilongjiang province, the altitude is 496 m and has a cold temperate continental monsoon climate. The variation of altitude and climate conditions leaded to the dissimilarity of local microbiota, finally giving rise to the distinct and highly diverse root-associated fungal/bacterial microbiomes. LZ belongs to the region of the Qinghai-Tibet Plateau, which is also characterized with low oxygen, reduced pathogen incidence, low temperature, and high levels of UV radiation (Qiao et al., 2016a, b). For plants, the plateau environment of high altitude, low pressure, low oxygen, strong radiation is undoubtedly harsh plant growth. Due to its unique natural and geographical environment, the microbial composition of *G. conopsea* in LZ is of great value. As shown in the results, *Leotiomycetes*, *Agaricomycetes*, *Eurotiomycetes*, and *Mortierellomycetes* were more abundant in LZ core fungal OTUs than DXAL in both root and soil samples at the vegetative growth stage, at family level, it includes *Helotiaceae*, *Dermateaceae*, *Vibrisseaceae*, *Myxotrichaceae and Herpotrichiellaceae*, which belongs to Dark septate endophytes (DSEs). DSEs belong to *Ascomycota* and widely distributed group of fungal root colonizers (Rodriguez et al., 2009). Previous researches revealed that DSEs can promote plant growth, possibly through nitrogen mineralization and absorption, and protection from pathogens (Mullen et al., 1998; Mandyam and Jumpponen, 2005; Newsham, 2011), DSEs have also been shown to positively influence plant drought resistance via enhancing nutrient and water uptake, plant growth, and/or improving plant resistance to oxidative stress (Perez-Naranjo, 2009; Santos et al., 2017). Many studies have shown that the plants that can live in the plateau are closely related to DSEs, which can enhance the growth of host plants and absorption of nutrients such as phosphorus (Jumpponen et al., 1998; Jumpponen, 2001). Arbuscular mycorrhiza (AM) is one member of an ancient and common mutualistic symbiosis, formed between *Glomeromycota* fungi and many plants, in which fungi supply plants of nutrients obtained from soil in exchange for lipids and carbohydrates (Delaux et al., 2015; Rich et al., 2017). In our results, *Ambisporaceae*, *Archaeosporaceae*, *Acaulosporaceae*, *Gigasporaceae*, *Glomeraceae* and *Paraglomeraceae* significantly enriched in LZ soil samples, most of these fungi are barely found in DXAL. In addition, *Clavariaceae*, *Russulaceae*, *Leotiaceae* and *Dictyosporiaceae* were more dominate in LZ root samples, whereas these families were rare in DXAL root samples. It indicated that these fungal taxa may play certain roles in adapting to specific environments in LZ. To summarize, these results implied that it might exist a different pool of fungal taxa in each site and the plant organized its microbiomes from these pools. However, although the microbial composition varied greatly from LZ to DXAL, the dominant taxa were similar, and just the relative abundance of taxa was different, implying that the symbiotic microorganisms of *G. conopsea* still have certain specificity.

As for the bacterial communities, *Acidobacteria* and *Actinobacteria* were significantly abundant in LZ root samples at two developmental stages, while *Actinobacteria*, *Planctomycetes* and *TM7* were enriched in LZ soil samples at two developmental stages. *Actinobacteria* were enriched in LZ from both root and soil samples compared with DXAL. *Actinobacteria* belongs to gram-positive saprophytic organisms and play an important role in plant development (Verma et al., 2009). Moreover, *Actinobacteria* have strong colonization ability and can live in a variety of soil, and spore formation which allows them to be able to survive longer at some extreme environments, such as irradiation, and drought (Qin et al., 2011; Yandigeri et al., 2012; Palaniyandi et al., 2013). *Acidobacteria* are abundant under various conditions with participation in sulfur cycling (Sanchez-Andrea et al., 2011; Serkebaeva et al., 2013; Urbanova and Barta, 2014), have the property that functionality of *Acidobacterial* dissimilatory sulfur pathways and degradation of cellulose produces acetic acid and hydrogen under anoxic condition (Pankratov et al., 2012; Hausmann et al., 2018). This result was consistent with the characteristics of *Acidobacteria* and the special environment of LZ.

## Conclusion

In addition to fungi, the roots of *G. conopsea* in wild also harbor a wide variety of bacteria, dominated by *Proteobacteria*, *Bacteroidetes*, *Acidobacteria*, *Actinobacteria*. The structure of root-associated microbiomes was significantly affected by geographical location in *G. conopsea*. Nevertheless, some taxa were observed in both locations, implying that these microbiomes have a widespread distributions that are not completely restricted to environmental conditions. Besides, the developmental stage and compartment were also factors contributing to microbiome variation. The richness of both fungal and bacterial communities was lower at reproductive growth stage than vegetative growth stage in the root samples from two locations, indicating that this species may be dependent on interaction with more fungi or bacteria during this early growth period. However, further investigations are needed to assess the variation in microbial community composition of root in *G. conopsea*, including samples from multiple different growth locations and more growth stages. Besides, we eagerly anticipate future studies that integrating multiple factors to explore the differentiation of microbial community composition in *G. conopsea*, such as variability on the genetic background and ploidy level. Overall, our study offers new clues to understand the microbiomes associated with two different locations and growth stages of an orchid species. Hopefully, these efforts could lay a basis for future microbiome-assisted *G. conopsea* cultivation.

## Data Availability Statement

All raw sequencing reads were deposited in the NCBI SRA database with the accession numbers PRJNA598558 (ITS data) and PRJNA598739 (16S data).

## Author Contributions

ZM conceived and supervised the project. ML collected samples and designed the experiment and wrote and revised the manuscript. ML and LL analyzed the data. HX, XX, and ZZ helped with sample treatment. LL provided comments and suggests on the results.

## Conflict of Interest

The authors declare that the research was conducted in the absence of any commercial or financial relationships that could be construed as a potential conflict of interest.
