# Supplementary material for: The Effect of Plant Geographical Location and Developmental Stage on Root-Associated Microbiomes of Gymnadenia conopsea
Source: Front Microbiol. 2020 Jun 18;11:1257. doi: 10.3389/fmicb.2020.01257 (PMC7314937; doi:10.3389/fmicb.2020.01257)
Supplement: Supplementary file 1 [file Data_Sheet_1.docx]

**Description of Supplementary Files**

File Name: Table S1

Description: Information of soil and root samples in two locations. LZ: Linzhi, DXAL: Greater Khingan Mountains. 1: vegetative growth stage, 2: reproductive growth stage.

File Name: Table S2

Description: Table displaying sequencing information for each sample in the ITS sequencing samples. LZ: Linzhi, DXAL: Greater Khingan Mountains. 1: vegetative growth stage, 2: reproductive growth stage.

File Name: Table S3

Description: Table displaying sequencing information for each sample in the 16S rRNA sequencing samples. LZ: Linzhi, DXAL: Greater Khingan Mountains. 1: vegetative growth stage, 2: reproductive growth stage.

File Name: Table S4

Description: PERMANOVA results using UniFrac as a distance metric for all experiments.

File Name: Table S5

Description: Relative abundances of different fungal/bacterial phyla in each group. DXAL: Greater Khingan Mountains, LZ: Linzhi, 1: vegetative growth stage, 2: reproductive growth stage.

File Name: Table S6

Description: Relative abundances of different fungal/bacterial classes in each group. DXAL: Greater Khingan Mountains, LZ: Linzhi, 1: vegetative growth stage, 2: reproductive growth stage.

File Name: Table S7

Description: Comparisons of classes differential abundance between compartments in each group from fungal communities. Hypothesis testing was carried out using Wilcoxon rank sum tests and corrected for multiple testing using the Benjamini-Hochberg method. DXAL: Greater Khingan Mountains, LZ: Linzhi, 1: vegetative growth stage, 2: reproductive growth stage.

File Name: Table S8

Description: Comparisons of classes differential abundance between compartments in each group from bacterial communities. Hypothesis testing was carried out using Wilcoxon rank sum tests and corrected for multiple testing using the Benjamini-Hochberg method. DXAL: Greater Khingan Mountains, LZ: Linzhi, 1: vegetative growth stage, 2: reproductive growth stage.

File Name: Table S9

Description: The relative abundance of fungal functional guild in each group. DXAL: Greater Khingan Mountains, LZ: Linzhi, 1: vegetative growth stage, 2: reproductive growth stage.

File Name: Table S10

Description: Reporter score for KO modules in bacterial microbiome. rs >0, soil-enriched; rs < 0, root-enriched. DXAL: Greater Khingan Mountains, LZ: Linzhi, 1: vegetative growth stage, 2: reproductive growth stage.

File Name: Table S11

Description: Relative abundances (‰) of different fungal classes in each group across different sites. Hypothesis testing was carried out using Wilcoxon rank sum tests and corrected for multiple testing using the Benjamini-Hochberg method, * represent < 0.05, ** represent < 0.01, *** represent < 0.001. DXAL: Greater Khingan Mountains, LZ: Linzhi, 1: vegetative growth stage, 2: reproductive growth stage.

File Name: Table S12

Description: Relative abundances (‰) of different bacterial phyla and families in each group across different sites. Hypothesis testing was carried out using Wilcoxon rank sum tests and corrected for multiple testing using the Benjamini-Hochberg method, * represent < 0.05, ** represent < 0.01, *** represent < 0.001. DXAL: Greater Khingan Mountains, LZ: Linzhi, 1: vegetative growth stage, 2: reproductive growth stage.

File Name: Table S13

Description: Relative abundances (‰) of different fungal classes in each group at different developmental stages. Hypothesis testing was carried out using Wilcoxon rank sum tests and corrected for multiple testing using the Benjamini-Hochberg method, * represent < 0.05, ** represent < 0.01, *** represent < 0.001. DXAL: Greater Khingan Mountains, LZ: Linzhi, 1: vegetative growth stage, 2: reproductive growth stage.

File Name: Table S14

Description: Relative abundances (‰) of different bacterial phyla and families in each group at different developmental stages. Hypothesis testing was carried out using Wilcoxon rank sum tests and corrected for multiple testing using the Benjamini-Hochberg method, * represent < 0.05, ** represent < 0.01, *** represent < 0.001. DXAL: Greater Khingan Mountains, LZ: Linzhi, 1: vegetative growth stage, 2: reproductive growth stage.

**File 2**

**Figure S1.** Microbial community composition among different sample types of *Gymnadenia conopsea* at class level. **(A)** Relative abundance of fungal taxa of each group. **(B)** Relative abundance of bacterial taxa of each group. DXAL: Greater Khingan Mountains, LZ: Linzhi, 1: vegetative growth stage, 2: reproductive growth stage

**Figure S2.** Comparative analyses of bacterial core microbiome across different sites. **(A)** Relative abundance (‰) of the phyla detected in soil core OTUs of the indicated plant growth sites at vegetative growth stage. **(B)** Relative abundance (‰ of families belonging to the three dominant phyla in the soil core OTUs of the indicated plant growth sites at vegetative growth stage. **(C)** Relative abundance (‰) of the phyla detected in soil core OTUs of the indicated plant growth sites at reproductive growth stage. **(D)** Relative abundance (‰ of families belonging to the three dominant phyla in the soil core OTUs of the indicated plant growth sites at reproductive growth stage. Asterisks indicate significant differences (Benjamini–Hochberg false discovery-rate (FDR) adjusted P value, * represent < 0.05, ** represent < 0.01, *** represent < 0.001).

**Figure S3.** Comparative analyses of bacterial core microbiome across different developmental stages. **(A)** Relative abundance (‰) of the phyla detected in soil core OTUs of the indicated developmental stages in Linzhi. **(B)** Relative abundance (‰ of families belonging to the three dominant phyla in the soil core OTUs of the indicated developmental stages in Linzhi. **(C)** Relative abundance (‰) of the phyla detected in soil core OTUs of the indicated plant growth sites in Greater Khingan Mountains. **(D)** Relative abundance (‰) of families belonging to the three dominant phyla in the soil core OTUs of the indicated plant growth sites in Greater Khingan Mountains. Asterisks indicate significant differences (Benjamini–Hochberg false discovery-rate (FDR) adjusted P value, * represent < 0.05, ** represent < 0.01, *** represent < 0.001).
