# Supplementary material for: The Effect of Plant Geographical Location and Developmental Stage on Root-Associated Microbiomes of Gymnadenia conopsea
Source: Front Microbiol. 2020 Jun 18;11:1257. doi: 10.3389/fmicb.2020.01257 (PMC7314937; doi:10.3389/fmicb.2020.01257)
Supplement: Supplementary file 3 [file Image_1.pdf]

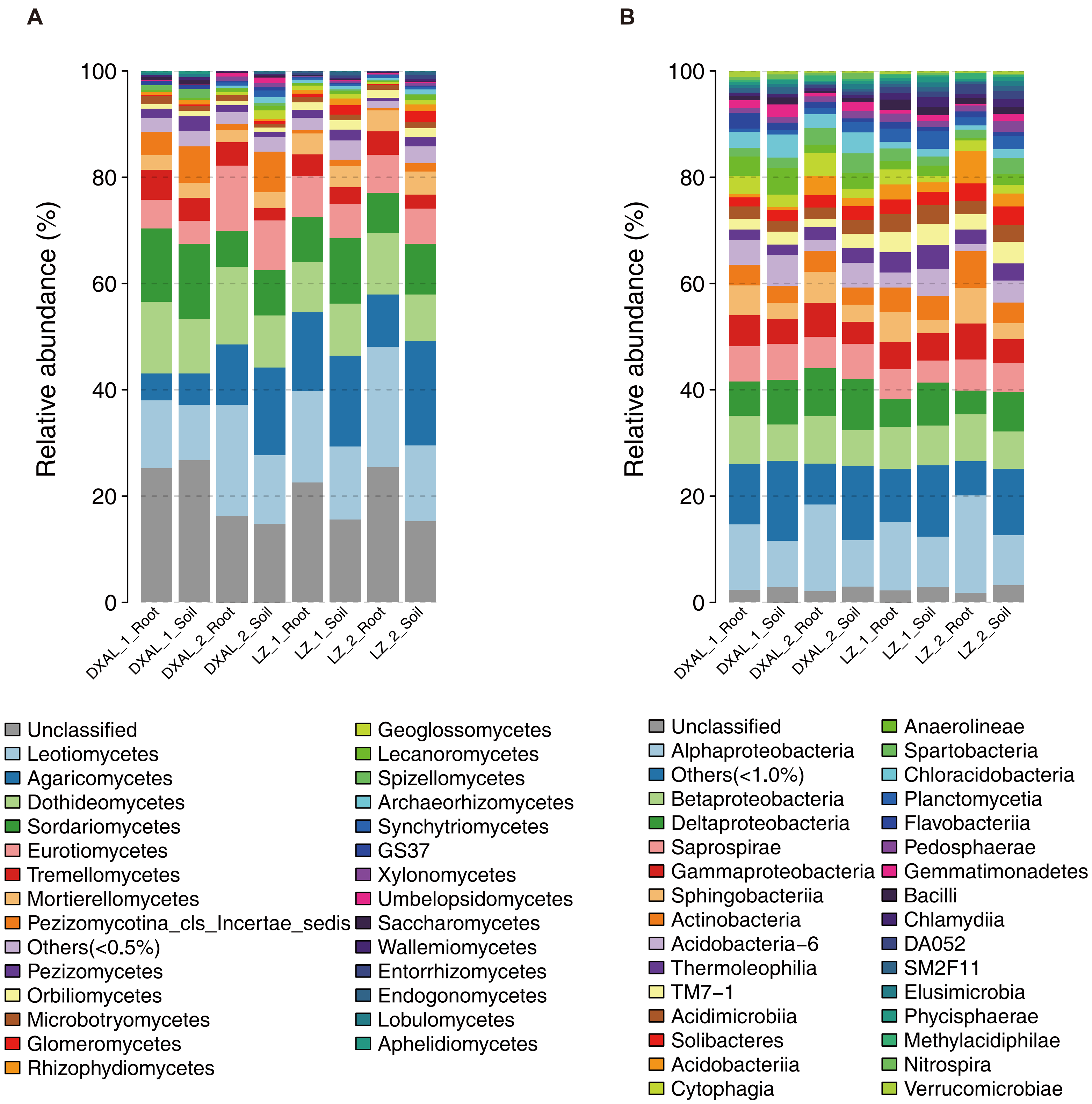
A
B
Relative abundance (%)
0
20
40
60
80
100
DXAL_1_Root
DXAL_1_Soil
DXAL_2_Root
DXAL_2_Soil
LZ_1_Root
LZ_1_Soil
LZ_2_Root
LZ_2_Soil
Unclassified
Leotiomycetes
Agaricomycetes
Dothideomycetes
Sordariomycetes
Eurotiomycetes
Tremellomycetes
Mortierellomycetes
Pezizomycotina_cls_Incertae_sedis
Others(<0.5%)
Pezizomycetes
Orbiliomycetes
Microbotryomycetes
Glomeromycetes
Rhizophydiomycetes
Geoglossomycetes
Lecanoromycetes
Spizellomycetes
Archaeorhizomycetes
Synchytriomycetes
GS37
Xylonomycetes
Umbelopsidomycetes
Saccharomycetes
Wallemiomycetes
Entorrhizomycetes
Endogonomycetes
Lobulomycetes
Aphelidiomycetes
Unclassified
Alphaproteobacteria
Others(<1.0%)
Betaproteobacteria
Deltaproteobacteria
Saprospirae
Gammaproteobacteria
Sphingobacteriia
Actinobacteria
Acidobacteria−6
Thermoleophilia
TM7−1
Acidimicrobiia
Solibacteres
Acidobacteriia
Cytophagia
Anaerolineae
Spartobacteria
Chloracidobacteria
Planctomycetia
Flavobacteriia
Pedosphaerae
Gemmatimonadetes
Bacilli
Chlamydiia
DA052
SM2F11
Elusimicrobia
Phycisphaerae
Methylacidiphilae
Nitrospira
Verrucomicrobiae
