# Supplementary material for: The Effect of Plant Geographical Location and Developmental Stage on Root-Associated Microbiomes of Gymnadenia conopsea
Source: Front Microbiol. 2020 Jun 18;11:1257. doi: 10.3389/fmicb.2020.01257 (PMC7314937; doi:10.3389/fmicb.2020.01257)
Supplement: Supplementary file 4 [file Image_2.pdf]

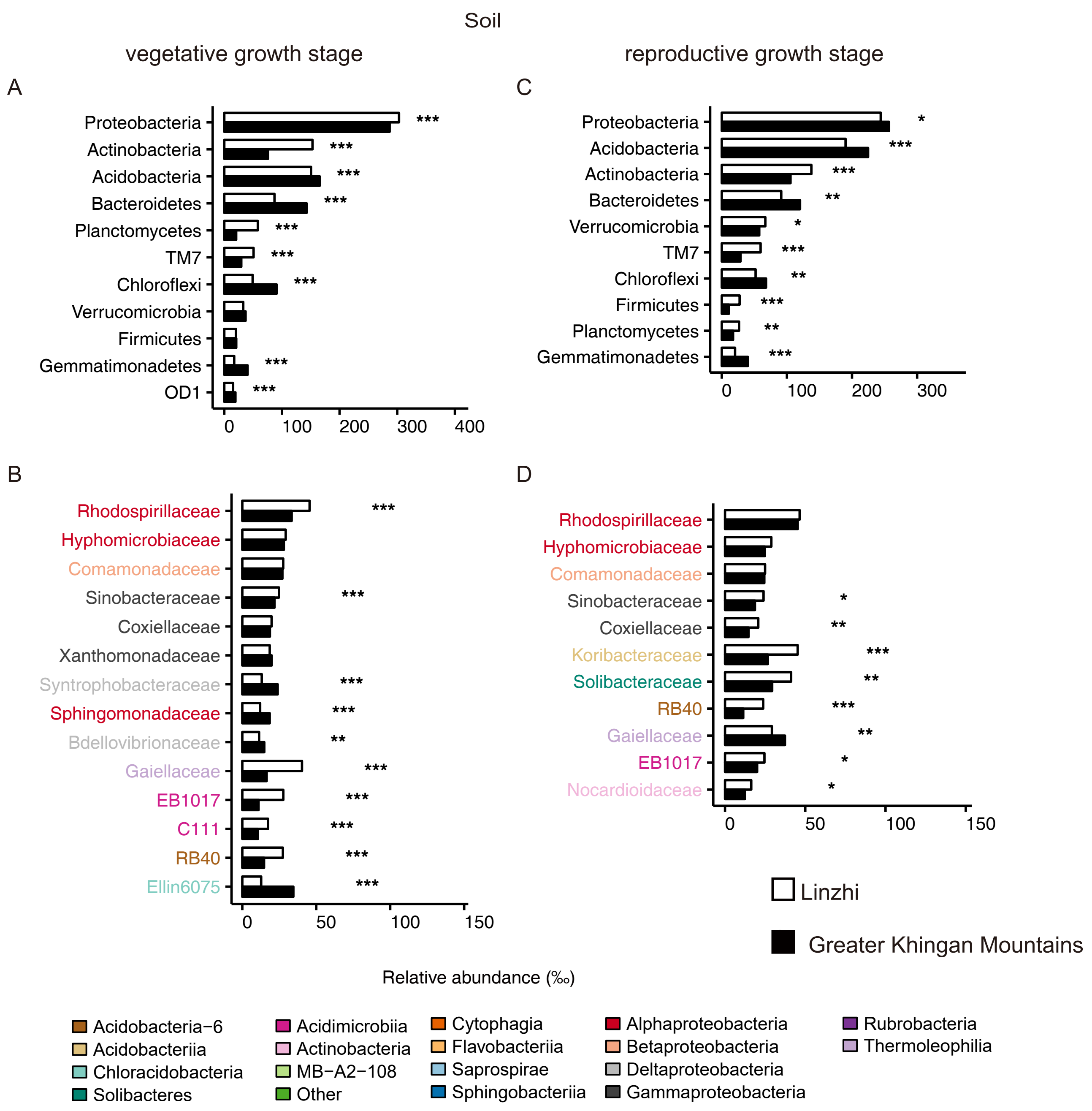

Soil
vegetative growth stage
reproductive growth stage
A
Proteobacteria
Actinobacteria
Acidobacteria
Bacteroidetes
Planctomycetes
TM7
Chloroflexi
Verrucomicrobia
Firmicutes
Gemmatimonadetes
OD1
0
100
200
300
400
C
Proteobacteria
Acidobacteria
Actinobacteria
Bacteroidetes
Verrucomicrobia
TM7
Chloroflexi
Firmicutes
Planctomycetes
Gemmatimonadetes
0
100
200
300
B
Rhodospirillaceae
Hyphomicrobiaceae
Comamonadaceae
Sinobacteraceae
Coxiellaceae
Xanthomonadaceae
Syntrophobacteraceae
Sphingomonadaceae
Bdellovibrionaceae
Gaiellaceae
EB1017
C111
RB40
Ellin6075
0
50
100
150
D
Rhodospirillaceae
Hyphomicrobiaceae
Comamonadaceae
Sinobacteraceae
Coxiellaceae
Koribacteraceae
Solibacteraceae
RB40
Gaiellaceae
EB1017
Nocardioidaceae
0
50
100
150
Linzhi
Greater Khingan Mountains
Relative abundance (‰)
Acidobacteria−6
Acidobacteriia
Chloracidobacteria
Solibacteres
Acidimicrobiia
Actinobacteria
MB−A2−108
Other
Cytophagia
Flavobacteriia
Saprospirae
Sphingobacteriia
Alphaproteobacteria
Betaproteobacteria
Deltaproteobacteria
Gammaproteobacteria
Rubrobacteria
Thermoleophilia
