# Supplementary material for: The Effect of Plant Geographical Location and Developmental Stage on Root-Associated Microbiomes of Gymnadenia conopsea
Source: Front Microbiol. 2020 Jun 18;11:1257. doi: 10.3389/fmicb.2020.01257 (PMC7314937; doi:10.3389/fmicb.2020.01257)
Supplement: Supplementary file 5 [file Image_3.pdf]

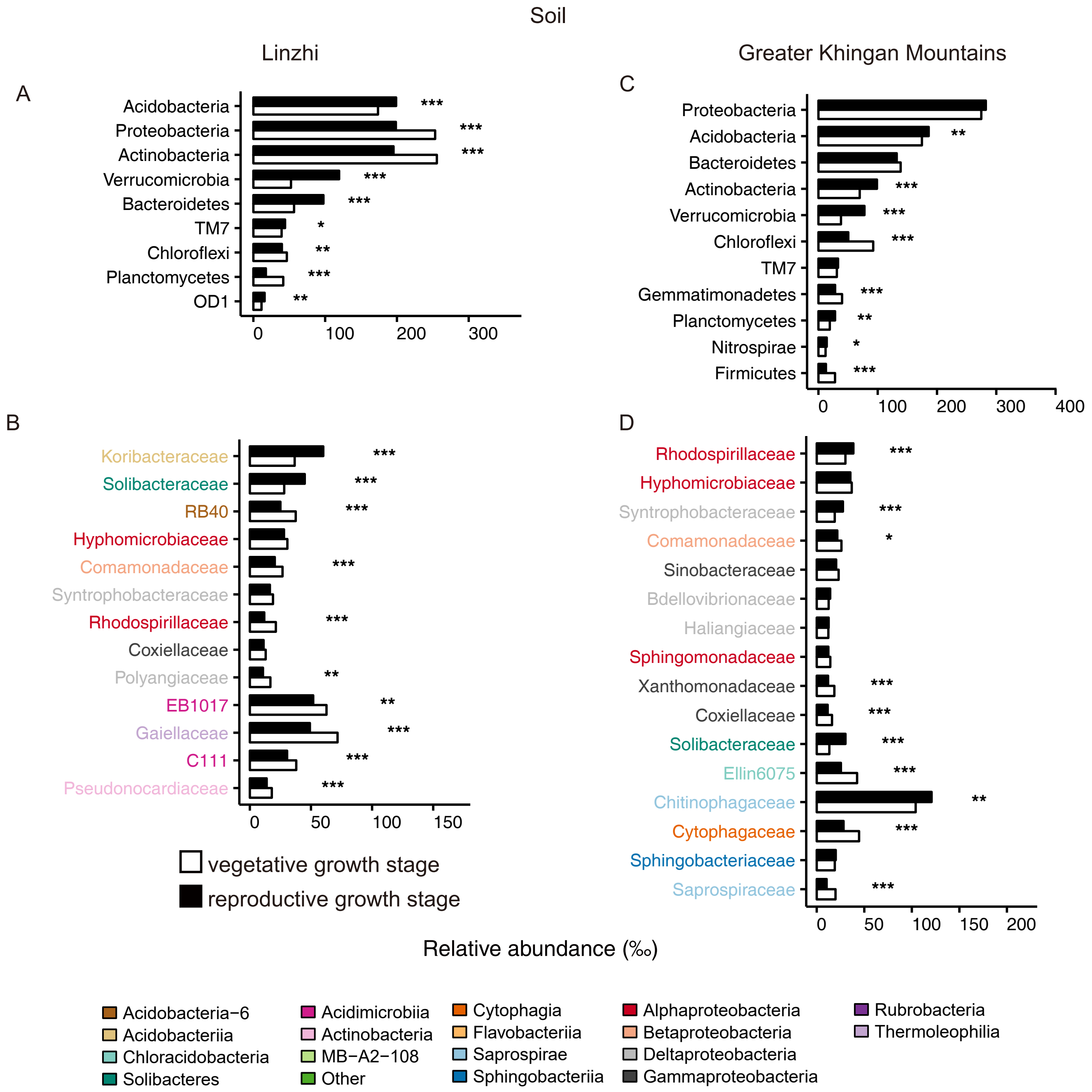

Soil
Linzhi
Greater Khingan Mountains
A
Acidobacteria
Proteobacteria
Actinobacteria
Verrucomicrobia
Bacteroidetes
TM7
Chloroflexi
Planctomycetes
OD1
0
100
200
300
C
Proteobacteria
Acidobacteria
Bacteroidetes
Actinobacteria
Verrucomicrobia
Chloroflexi
TM7
Gemmatimonadetes
Planctomycetes
Nitrospirae
Firmicutes
0
100
200
300
400
B
Koribacteraceae
Solibacteraceae
RB40
Hyphomicrobiaceae
Comamonadaceae
Syntrophobacteraceae
Rhodospirillaceae
Coxiellaceae
Polyangiaceae
EB1017
Gaiellaceae
C111
Pseudonocardiaceae
0
50
100
150
vegetative growth stage
reproductive growth stage
D
Rhodospirillaceae
Hyphomicrobiaceae
Syntrophobacteraceae
Comamonadaceae
Sinobacteraceae
Bdellovibrionaceae
Haliangiaceae
Sphingomonadaceae
Xanthomonadaceae
Coxiellaceae
Solibacteraceae
Ellin6075
Chitinophagaceae
Cytophagaceae
Sphingobacteriaceae
Saprospiraceae
0
50
100
150
200
Relative abundance (‰)
Acidobacteria−6
Acidobacteriia
Chloracidobacteria
Solibacteres
Acidimicrobiia
Actinobacteria
MB−A2−108
Other
Cytophagia
Flavobacteriia
Saprospirae
Sphingobacteriia
Alphaproteobacteria
Betaproteobacteria
Deltaproteobacteria
Gammaproteobacteria
Rubrobacteria
Thermoleophilia
